# Increased Fibroblast Metabolic Activity of Collagen Scaffolds via the Addition of Propolis Nanoparticles

**DOI:** 10.3390/ma13143118

**Published:** 2020-07-13

**Authors:** Jeimmy González-Masís, Jorge M. Cubero-Sesin, Yendry R. Corrales-Ureña, Sara González-Camacho, Nohelia Mora-Ugalde, Mónica Baizán-Rojas, Randall Loaiza, José Roberto Vega-Baudrit, Rodolfo J. Gonzalez-Paz

**Affiliations:** 1 Centro de Investigación y Extensión en Materiales, Escuela de Ciencia e Ingeniería de los Materiales, Instituto Tecnológico de Costa Rica, 159-7050 Cartago, Costa Rica; jeimygonz@gmail.com (J.G.-M.); jcubero@itcr.ac.cr (J.M.C.-S.); 2 Adolphe Merkle Institute, University of Fribourg, Chemin des Verdiers 4, 1700 Fribourg, Switzerland; yendry386@hotmail.com; 3 Biological Assay Laboratory (LEBi), Universidad de Costa Rica, 11501-2060 San José, Costa Rica; sara.gonzalez@ucr.ac.cr; 4 National Center for Biotechnological Innovations (CENIBiot), National Center of High Technology (CeNAT-CONARE), Pavas, 1174-1200 San José, Costa Rica; none.0512@gmail.com (N.M.-U.); mbaizan@cenat.ac.cr (M.B.-R.); rloaiza@cenat.ac.cr (R.L.); 5 National Laboratory of Nanotechnology (LANOTEC), National Center of High Technology (LANOTEC-CeNAT-CONARE), Pavas, 1174-1200 San José, Costa Rica; jvega@gmail.com; 6 Laboratorio de Polímeros (POLIUNA), Universidad Nacional, 86-3000 Heredia, Costa Rica

**Keywords:** propolis, solubility, dispersion, regenerative medicine

## Abstract

Propolis natural extracts have been used since ancient times due to their antioxidant, anti-inflammatory, antiviral, and antimicrobial activities. In this study, we produced scaffolds of type I collagen, extracted from Wistar Hanover rat tail tendons, and impregnated them with propolis nanoparticles (NPs) for applications in regenerative medicine. Our results show that the impregnation of propolis NPs to collagen scaffolds affected the collagen denaturation temperature and tensile strength. The changes in structural collagen self-assembly due to contact with organic nanoparticles were shown for the first time. The fibril collagen secondary structure was preserved, and the D-pattern gap increased to 135 ± 28 nm, without losing the microfiber structure. We also show that the properties of the collagen scaffolds depended on the concentration of propolis NPs. A concentration of 100 μg/mL of propolis NPs with 1 mg of collagen, with a hydrodynamic diameter of 173 nm, was found to be an optimal concentration to enhance 3T3 fibroblast cell metabolic activity and cell proliferation. The expected outcome from this research is both scientifically and socially relevant since the home scaffold using natural nanoparticles can be produced using a simple method and could be widely used for local medical care in developing communities.

## 1. Introduction

Recently, a vast amount of work has been carried out in regenerative medicine that has focused on generating various types of scaffolds and dressings loaded with bioactive compounds. Collagen is commonly used as a matrix material because it adheres to the skin easily, its use is simple, it is biocompatible, and it does not need to be removed [1]. Naturals extracts have been impregnated into collagen-based materials, not only due to their biocompatibility and low toxicity but also due to their capability to treat particular conditions [2,3]. However, lipophilic natural extracts are difficult to disperse in water, and consequently, to impregnate homogeneously within the collagen. Propolis is a natural lipophilic waxy resin extracted from beehives and is produced by honeybees as glue to seal the hive and protect it against pathogens, such as bacteria, viruses, and fungi [4,5]. This natural resin contains bioactive compounds that allow it to be used as an antiviral [4], antibacterial [5], antiseptic [5], food preservative [6], antioxidant [7], antimicrobial [8,9,10,11], anti-inflammatory [6], antifungal [12], immunostimulant, and antitumor agent [13]. Lately, propolis has been used as an agent for dental pulp tissue regeneration [5,7], increasing the collagen cell expression during wound healing [14,15], improving tissue repair [16], and as a bioactive nanocarrier [17]. The chemical composition of propolis is variable and complex since it depends on the flora of the geographic origin, particularly the collection area. Flavonoids, polyphenols, wax, essential oils, bee pollen, minerals, fatty acids, prenylated p-coumaric acids, caffeoylquinic acids, lignans, diterpenic acids, triterpenes, steroids, and sugars have been identified in propolis extracts of different origins [18,19]. Flavonoids and phenolic compounds are the main bioactive compounds [20]. These properties make propolis an excellent candidate for the modification of biocompatible materials, such as collagen scaffolds. We hypothesized that the impregnation of a collagen scaffold with an aqueous formulation containing propolis nanoparticles (NPs) could provide a homogeneous dispersion on the surface, increasing its bioactivity and also improve the ability of the bioactive compounds contained inside the NPs to cross the cell membrane [18]. This study aimed to prepare and characterize collagen scaffolds impregnated with propolis nanoparticles and to study their bioactivity regarding 3T3 fibroblast cell growth for a potential application in tissue engineering. The nanoparticles and scaffolds were characterized using various techniques, namely, dynamic light scattering (DLS), Fourier-transform infrared spectroscopy (FT-IR), atomic force microscopy (AFM), and differential scanning calorimetry (DSC).

## 2. Materials and Methods

**Figure 1:**
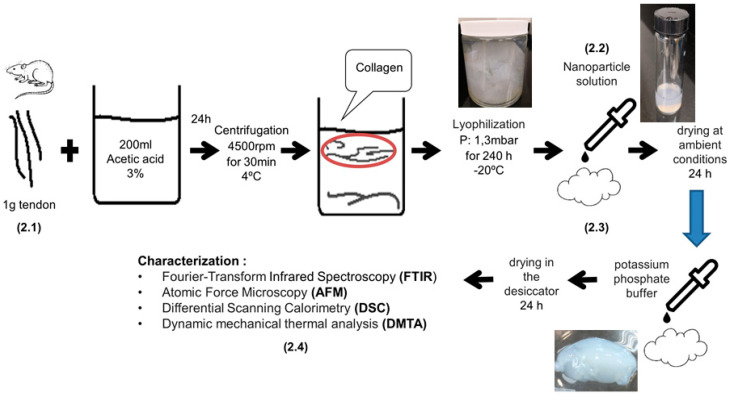
Schematic of experimental procedures followed during the preparation of collagen scaffolds impregnated with propolis nanoparticles and the corresponding characterization techniques.

Figure 1 shows a schematic of the experimental procedures followed for the preparation of the collagen scaffolds impregnated with propolis nanoparticles, as described in detail in Section 2.1, Section 2.2 and Section 2.3. The thermal, morphological, compositional, mechanical, and cyto-toxicological characterization is described in Section 2.4.

### 2.1. Type I Collagen Extraction

Tendons from Wistar Hannover male specimens were provided by the Laboratorio de Ensayos Biológicos (LEBi) from Universidad de Costa Rica. Type I collagen was extracted from the tendons under the following procedure: 1 g of tendon was solubilized in 200 mL of 3% acetic acid solution for 24 h while stirring. The temperature was kept constant at 4 °C. The solution was then filtered using gauze at room temperature and centrifuged at 4500 rpm for 30 min (ROTO SILENTA 630 RS, Hettich, Kirchlengern, Germany). The supernatant was lyophilized for 240 h at 1.3 mbar and −20 °C (Martin Christ beta 1–8 LSC, Osterode am Harz, Germany). Type I collagen from rat tail tendons is commonly used in tissue engineering applications according to well-standardized protocols. The high purity of the collagen makes it suitable for investigating structural changes in microfibers during self-assembly in the presence of organic propolis nanoparticles [21].

### 2.2. Propolis Extraction Process and NP Formation

Propolis from *Apis mellifera* bees was donated by local beekeeper farmers from Puriscal, Costa Rica. The propolis raw material was scraped from the hive’s surface. The following procedure was followed to produce the alcoholic extracts: 0.61 g of propolis raw material was diluted in 8 mL of 96% ethanol while stirring, before filtration at 4 °C with 90 mm grade 1 filter paper (Whatman, GE Healthcare, Chicago, IL, USA). A homogeneous, brown-colored, alcoholic extract was obtained. The NPs were produced following a previously published protocol [22]: 5 drops of 20 μL each of the alcoholic extract were added to 10 mL of MilliQ water (Milli-Q System, Merck-Millipore, Darmstadt, Germany) while stirring. The final ethanol concentration was 1% v/v. An ethanol evaporation step was carried out to decrease the ethanol volume. The hydro-alcoholic extract was placed in an oven at 45 °C for 16 h. The hydro-alcoholic suspension was sonicated for 5 min. The ultrasonic bath conditions were: 4 W, 40% amplitude, and a 6 mm probe diameter. Finally, the suspensions were filtered using a 0.2 μm sterile filter (Sartorius, Göttingen, Germany).

### 2.3. Preparation of Collagen Scaffolds Impregnated with Propolis NPs

The lyophilized collagen was impregnated with the propolis nanoparticle suspension at an amount of 100 μL per 1 mg of collagen. Films of collagen–propolis NPs were dried under ambient conditions for 24 h. The films were re-hydrated with PBS 0.1 M in the proportion of 100 μL per 1 mg of collagen. The films were dried one more time for the characterization.

### 2.4. Total Polyphenol Content

Solutions with concentrations of 100, 50, 10, 5, and 1 μg/mL were prepared for the determination of the calibration curve. A mixture of 0.75 mL of distilled water, 0.5 mL of the solutions, and 0.625 mL of the Folin reagent (1 N) were placed in a volumetric flask of 5 mL. Then, the flask was filled with a solution of Na_2_CO_3_ 10H_2_O (20% m/v). The mixtures were incubated for 40 min at room temperature in the dark. After the reaction period, the absorbance of the solutions was measured at a wavelength of 725 nm in a Shimadzu UV-Vis spectrophotometer (model UV-1800, Shimadzu Corporation, Kyoto, Japan).

### 2.5. Chemical and Physical Characterization of Collagen-Propolis NP Scaffolds

Fourier-transform infrared spectroscopy (FTIR): A Nicolet 6700 FTIR spectrophotometer (Thermo Scientific, Waltham, MA, USA) was used, scanning through wavenumbers from 4000 to 400 cm^−1^ with a standard resolution of 0.09 cm^−1^ and a scanning speed of 32 cm^−1^/s.

Differential scanning calorimetry (DSC): Thermal properties were measured on a Q200 DSC (TA Instruments, Mettler Toledo, Columbus, OH, USA) using a temperature ramp of 10 °C/min with scans over a range of 20–200 °C. For the analysis, 5 mg of the material was placed on an aluminum pan. Each measurement was repeated at least three times.

Amplitude-modulated atomic force microscopy (AFM): The re-hydrated scaffolds were dried in environmental conditions on glass coverslips. The sample topography was analyzed using an AFM operated in tapping mode (Asylum Research, Santa Barbara, CA, USA) in air. Silicon probes (model Tap150Al-G (Budget Sensors, Sofia, Bulgaria), with Al-covered cantilever backsides) with a resonance frequency of 150 kHz and force constant of 5 N/m were used. A scanning force microscope (SFM) operated in tapping mode (Digital Instruments Nanoscope III multimode SPM, Santa Barbara, CA, USA) in air and Nanoscope software (version 5.31R1) were used for acquiring the height and phase images.

Dynamic mechanical thermal analysis (DMTA): The thermo-mechanical properties of the collagen films were measured in tension at room temperature (25 °C) using a Q500 (TA Instruments, Newcastle, UK) with a testing strain of 10%, a gap load of 65 mm, a clamp face of 4.5 mm, and a gap speed of 16.66 μm/s. Tensile specimen dimensions were between 4.5–5 mm in width, 27–29 mm in length, and 1.3 mm in thickness.

Dynamic light scattering (DLS) and zeta potential: The size distribution and zeta potential were measured using a Zetasizer (Nano ZS90, Malvern Panalytical, Malvern, UK) at λ1 = 633 nm. Nanoparticle solutions of 0.1 mg/mL were analyzed.

### 2.6. Cellular Response to Propolis-Enriched Collagen Scaffolds: Cell Viability and Proliferation

Fibroblast cell culture: A 3T3 mouse fibroblast cell line (ATCC, Manassas, VA, USA) was maintained and cultured in standard conditions (37 °C, 5% CO_2_) in Dulbecco’s Modified Eagle Medium (DMEM). The medium was supplemented with 10% fetal bovine serum and changed every other day. The cells were sub-cultured using 0.25% (*w*/*v*) Trypsin 0.53 mM EDTA (Sigma-Aldrich, St. Louis, MO, USA) while keeping the confluency at 25–80%.

Cell viability assay: The test was performed following standard protocols [23]. Polypropylene 96-well plates without a surface treatment for enhancing cell adhesion were used (Greiner Bio One, Frickenhausen, Germany). The 3T3 mouse fibroblast cell line in suspension with 5 × 10^4^ cells/well was pipetted onto wells in triplicate with a corresponding medium-only well (fluorescence blank) containing a collagen matrix with propolis NPs at 10, 100, and 1000 μg/mL; a collagen matrix without the nanoparticles; and culture medium for 48 h. Propolis NPs at these concentrations were previously evaluated following the same protocol and cell line [22]. The fluorescence was measured with a staining assay based on resazurin salts (Sigma-Aldrich, R7017, St. Louis, MO, USA) to calculate the cell viability. Fluorescence readings were performed at 8, 24, and 48 h using a microplate reader (Synergy H1 Hybrid, Biotek, Winooski, VT, USA) with a gain of 70, an excitation wavelength of 540 nm, and an emission wavelength of 590 nm. The fluorescence blank was subtracted from the average of the data (supernatant with a culture medium in the absence of cells). The percentage of viability was calculated as shown in Equation (1):

(1) $$\mathrm{Viability}\;\left(\%\right)=\frac{\mathrm{Average}\;\mathrm{of}\;\mathrm{cells}\;\mathrm{with}\;\mathrm{treatment}}{\mathrm{Average}\;\mathrm{of}\;\mathrm{cells}\;\mathrm{without}\;\mathrm{treatment}}\times 100.$$

Cell proliferation assay: A 3T3 mouse fibroblast cell line in suspension with 5 × 10^4^ cells/well was pipetted onto wells in triplicate with a corresponding medium-only well (fluorescence blank) containing a collagen matrix with propolis NPs at 10, 100, and 1000 μg/mL; a collagen matrix without the nanoparticles; and a culture medium for 48 h 100 μL of 0.25% trypsin/EDTA solution was added to each well. The plate was incubated at 37 °C while stirring for 15 min. After the incubation period, 100 μL of 10% fetal bovine serum (FBS) in Dulbecco’s Modified Eagle Medium (DMEM) was added to inhibit the trypsin. The cell suspension was centrifuged at 3500 rpm. The formed pellet was resuspended in 10 μL of culture medium. Trypan Blue 0.4% staining solution (Sigma-Aldrich, Darmstadt, Germany) was used to stain the dead cells, and consequently, to quantify the living cells. A total of 10 μL of Trypan Blue 0.4% was gently mixed with 50 μL of the cell suspensions. Finally, 50% of the cell suspension was placed on a counting slide (Bio-rad, Hercules, CA, USA) and the cells were counted using a TC20 automated cell counter (Bio-Rad, USA).

Statistical methods: The experiments were repeated five times each and replicated in triplicate. The statistical analysis was performed in RStudio (Version 1.2.5001, Rstudio Inc, Boston, MA, USA)

## 3. Results

**Figure 2:**
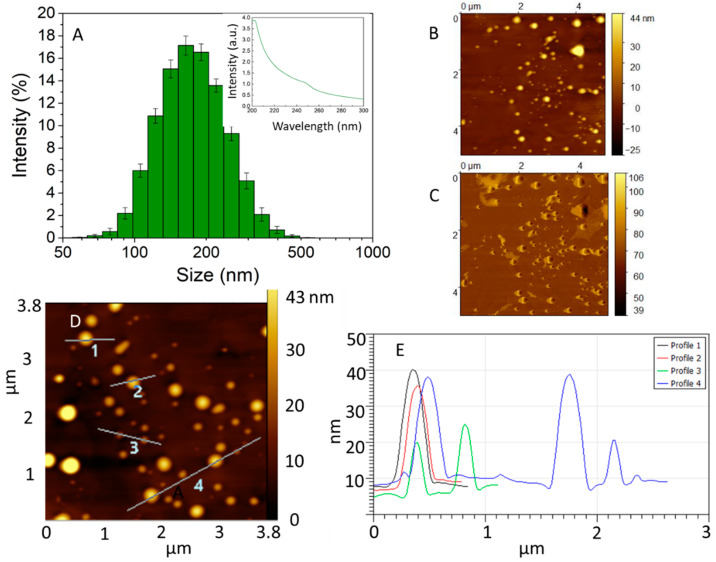
(**A**) Dynamic light scattering (DLS) data of dispersions of 1 mg/mL propolis showing the main NP population between 70–300 nm. Inset: UV-vis spectra of the propolis ethanolic extract showing an absorption band at 250 nm. AFM image of (**B**) the topography and (**C**) the phase of NPs adsorbed on highly pyrolytic oriented graphite. (**D**) AFM topography image showing line profiles. (**E**) Cross-section height derived from the line profiles shown in (**D**); nanoparticles between 3 to 40 nm height were imaged.

An extract rich in polyphenols was obtained from the raw propolis using ethanol as a solvent. This solvent was selected because of its low toxicity to cells in controlled amounts, good polyphenol solubility, and its previous use in the food industry to extract propolis [24]. The amount of polyphenols in the alcoholic extract was quantified using the Folin–Ciocalteu method [22]. A concentration of 9.38 ± 1.24 mg/mL was estimated. The presence of polyphenolics in the extract was confirmed due to the absorption band at 250 nm, which is characteristic of aromatic compounds, as shown in the inset of Figure 2A. The propolis extract was partially soluble in water and tended to form micellar aggregates when it was dissolved in the aqueous media. To form the NPs, ultrasound was applied during the pipetting of the ethanolic extract to water to generate shear stress, increase the local temperature, and decrease the size of the propolis aggregates. The suspension obtained was brownish and opaque. The hydrodynamic diameter of the obtained propolis particles was measured using the DLS technique (hydrated state) and AFM (semi-dried state) [22]. The histogram in Figure 2A shows the distribution of the particle sizes, where the average was determined to be 174.6 ± 63 nm. The obtained polydispersity was 0.11 and the Z potential was −41.2 ± 9. These results suggest a stable suspension. Figure 2B,D shows representative AFM topography images displaying particles with a pancake-like morphology, which is characteristic of soft particles adsorbed on a surface [25]. A low amount of organic material was covering the substrate and surrounding the particles, as can be seen in the phase image from Figure 2C. Therefore, line profiles of areas where the particles were adsorbed, as indicated in Figure 2D, indicated particles that were between 3 nm and 40 nm in height, according to Figure 2E.

**Figure 3:**
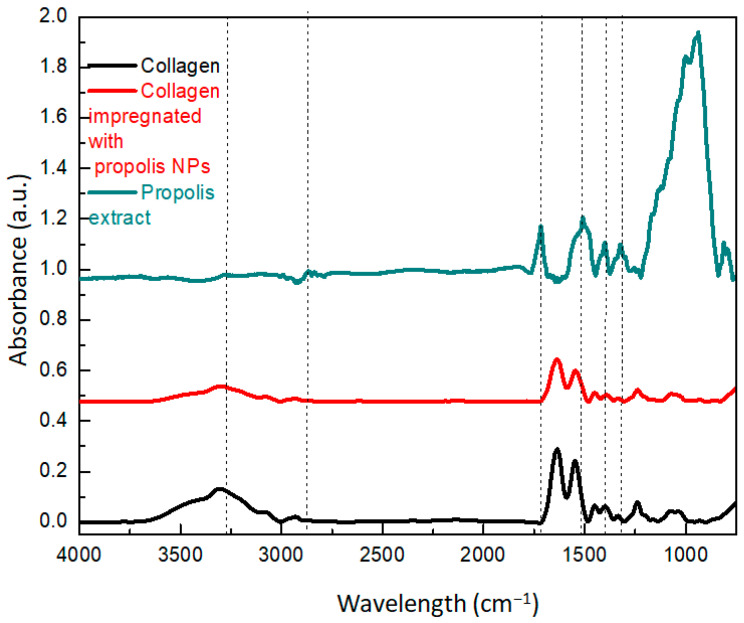
FTIR spectra of the collagen, ethanolic extract, and collagen scaffolds impregnated with propolis NPs.

**Figure 4:**
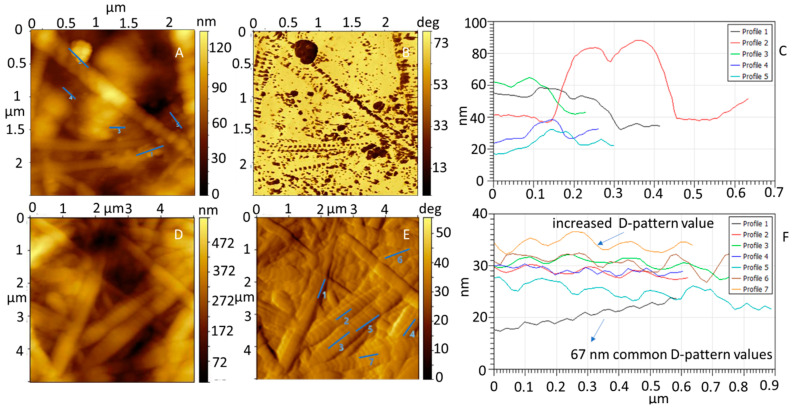
(**A**) AFM topography and (**B**) phase image of collagen impregnated with propolis NPs. (**C**) Cross-section of the line profiles shown in (**A**). (**D**) AFM topography image of collagen impregnated with propolis NPs. (**E**) Amplitude image of the topography image shown in (**D**). (**F**) Cross-section of the line profiles shown in (**E**). The collagen D-pattern gap was increased.

The FTIR spectra of the collagen, propolis extract, and collagen–NPs are shown in Figure 3. A characteristic band at 3270 cm^−1^ corresponded to phenolic compounds [26], whereas the small peak at 2866 cm^−1^ was associated with CH_2_ groups of alkyl compounds. The peak at 1720 cm^−1^ was attributed to axial stretches of carbonyl aliphatic ketone [27]. The peaks between 1100 and 1600 cm^−1^ could be correlated to flavonoids [28]. Specifically, the peak at 1508 cm^−1^ was attributed to the stretching vibration of the C=O bonds that are characteristic of caffeic acid and its derivatives [28]. The band at 1500 cm^−1^ was associated with C–C rings occurring in pairs (at 1600 and 1500 cm^−1^) and were more prominent in the nanoparticle spectrum. The bands at 1040 and 9370 cm^−1^ were associated with C–O ester groups and C–H aromatic rings, respectively [27]. The peak at 717 cm^−1^ was attributed to the stretching vibration of the carbonyl C=O bonds, which are characteristic of caffeic acid and its derivatives [11]. The propolis extract did not show peaks at 540 and 630 cm^−1^ that correspond to amide bonds of proteins, and consequently, the UV absorption band at 225 and 270 nm could be associated with flavonoids and phenols [28]. The impregnation protocol was chosen to avoid phase separation or aggregation of the propolis compounds due to the differences in hydrophobicity between the particles and the collagen matrix. Furthermore, it allowed the collagen to self-assemble in its characteristic fibrils and bundles to maintain the collagen scaffold’s mechanical properties and to preferentially expose the particles on the surface. The FTIR results suggested that the NP impregnation did not change the interaction between the protein molecules since the amide I and amide II peaks did not shift. This type of fibril structure in the nano, micro, and macro scales plays a fundamental role in the maintenance of the thermal stability and physical and functional characteristics of the collagen [17]. The propolis is called bee-glue because it is a soft and very sticky material. Polyphenol derivatives are natural chelators and they form strong bonds with proteins through intra/intermolecular H-bonds [29]. The results suggest that the propolis nanoparticles did not disrupt the assembly of collagen fibers and mainly interacted with their surface or in between the fibrils, as confirmed by the AFM analysis from Figure 4.

The collagen control matrix and the collagen matrix impregnated with NPs showed a fibrillar structure, according to the AFM height images in Figure 4A,D. In Figure S1, the collagen without NPs shows a similar structure. Single-NP dispersion of propolis on the surface was verified by comparing the phase and topography images of the collagen with propolis NPs in Figure 4A,B with the respective images of the collagen in Figure S1A,B. The surface of the collagen impregnated with propolis NPs showed round particles distributed on the surface and having a different adhesion to the AFM tip, as shown from the phase map analysis in Figure 4B,C. There was a correlation between the places where the nanoparticles were located and the changes in the phase image (darker contrast). The size of the particles was determined to be 27 ± 8 nm.

**Figure 5:**
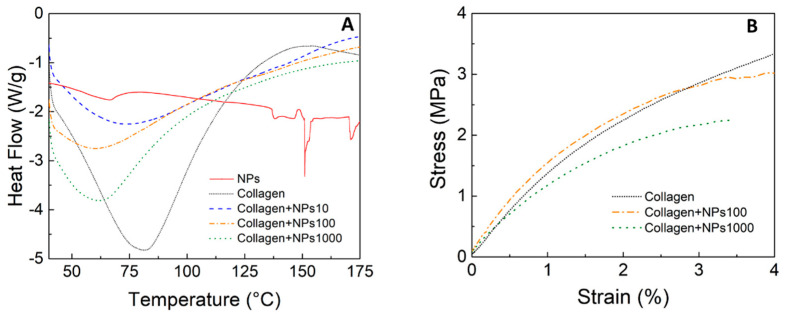
(**A**) DSC curves for type I collagen, propolis NPs, and collagen impregnated with propolis nanoparticles at different concentrations. (**B**) Stress–strain diagram of collagen impregnated with propolis nanoparticles at different concentrations.

The fibril structure was maintained after the impregnation with the nanoparticles. However, changes in the D-pattern gap from 67 ± 5 nm to 135 ± 28 nm were detected based on the analysis of Figure 4F. A pattern of dark contrast spots along the collagen fibrils can be seen in the areas where the gaps are located. This result indicates that the changes could be produced by the deposition of propolis NPs on and in between collagen fibrils during the microfiber self-assembly. Strong interaction in these areas could be promoted due to the enhanced probability of hydrogen bond formation and electrostatic interactions triggered by the higher density of C and N peptidic chain termini [30]. Therefore, changes in the amount of water adsorbed (retained bonded active water) could occur due to changes in the fibril surface chemistry [31]. The change in the D-pattern gap was reproducible; small variations of ±8 nm between four of the independent samples were obtained. To determine the morphological integrity of the collagen scaffolds with various concentrations of propolis NPs, the denaturation temperature and the thermal stability (in terms of the enthalpy of the denaturation reaction in the collagen matrix) were analyzed using DSC. The results are shown in Table 1 and Figure 5A. An additional DSC from the NP solution is included in Figure 5A as a reference.

The impregnation of propolis NPs generated changes in the denaturation temperature of the collagen. A denaturation temperature of 81.52 °C was observed for the self-assembled collagen, which is consistent with studies carried out by other researchers [32]. At this point, the crystalline triple helical structure of the collagen became an amorphous random spiral due to the variations that occurred in the intermolecular bonds between each triplet (–Gly–xy–) of a given chain that joined two other nearby chains through two hydrogen bonds [33]. Furthermore, a very slight inflection was observed near 150 °C, which was related to the melting of propolis [34]. The collagen containing a concentration of 10 μg/mL of ≈77 °C, which was a similar denaturation temperature to collagen alone. However, increasing the NP concentration decreased the denaturation temperature to ≈63 °C. This result indicates that the nanoparticles affected the interaction between the collagen molecules and that there was an increase in the polypeptide chain mobility. The higher degree of crosslinking in the collagen resulted in a higher temperature required to produce the molecular movement that increased the entropy of the triple helices, which led to denaturation [35]. Furthermore, this was reflected in the lower energetic cost for denaturing the fibers, which was directly correlated with the enthalpy values that dropped from 326 J/g to 209.8 J/g.

Concerning the mechanical properties, Figure 5B shows the stress–strain curves for the collagen and the collagen + propolis NPs. The elongation for the applied stress showed a typical viscoelastic behavior. Both the tensile strength and the elongation to failure were higher for the collagen than for the collagen impregnated with the propolis NPs, showing that the NPs influenced the crosslinked state of the collagen. A higher NP concentration caused a lower tensile strength. These results confirm the importance of testing the integrity of the mechanical properties of the material when adding organic nanoparticles, even in low concentrations. The differences in cross-linking affected not only the tensile strength but also the dissolution in different media. González et al. previously showed an increase in the cell viability of 3T3 fibroblasts in contact with 10, 100, and 1000 μg/mL solutions of propolis NPs [22].

**Figure 6:**
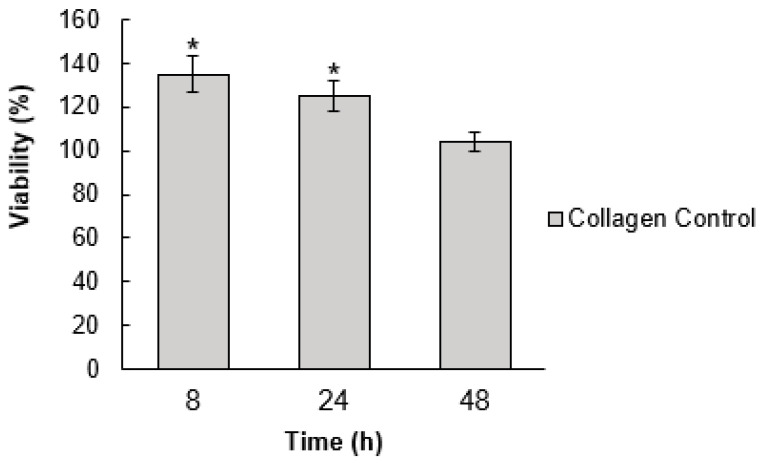
Cell viability of a 3T3 cell line incubated with collagen for 8, 24, and 48 h. The values were normalized with the results obtained for cells growing in culture media (mean % ± SD). Significant differences at *p* ≤ 0.05 are indicated by an asterisk (*). Significance was determined using an analysis of variance (ANOVA) test.

The effect of the pristine collagen matrix on the cell viability was initially determined at 8, 24, and 48 h. A significant difference was observed between the cells growing in culture media and the collagen at 8 and 24 h (Figure 6). According to the data reported in the literature, the pristine collagen tends to increase cell viability with time [36,37,38]. However, we did not observe an enhancement in the cell viability using the collagen from the rabbit tail tendon. It is important to highlight that this collagen was mainly chosen because of its simple extraction process and high purity, which allowed for a clear structural characterization. The time of 48 h was chosen for studying the nanoparticle effect due to the minimal effect on cell viability.

**Figure 7:**
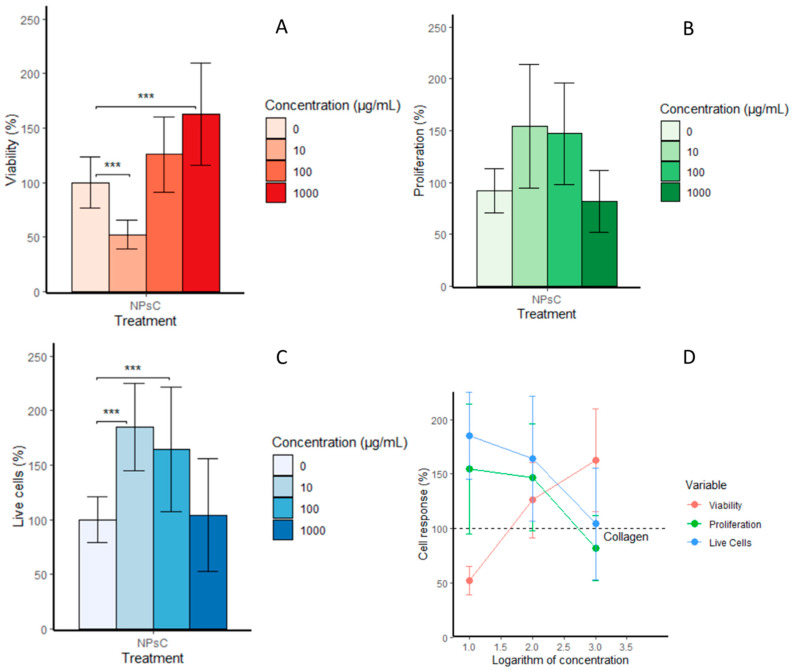
(**A**) Cell viability of 3T3 cell line incubated with collagen and collagen impregnated with propolis NPs at concentrations of 10, 100, and 1000 μg/mL (mean % ± SD). (**B**) Total cell count means determined using a proliferation assay. (**C**) Number of living cells. (**D**) Correlation of cell response and propolis NP concentration. Significant differences at *p* ≤ 0.05 are indicated by a line and asterisks (***). Significance was determined using an analysis of variance (ANOVA) test.

Figure 7A shows the results of the cell viability assay. A significant increase in the viability was observed for the highest concentration of 1000 μg/mL when compared to the viability of the collagen control sample for 48 h. The cell response showed an increase in viability in a dose-dependent manner, as shown in Figure 7D. However, the higher concentration also decreased the cell proliferation and percentage of living cells, as seen in Figure 7B,C. There was an enhancement in cell proliferation for the concentrations of 100 and 10 μg/mL but there was no significant difference between the collagen impregnated with these two concentrations and the pristine collagen. The percentages of living cells obtained for 10 and 100 μg/mL were higher and significantly different compared to the control sample (*p* ≤ 0.05), which suggests that the increase in the viability could have been mainly due to a metabolic stimulus provided by the propolis NPs. The positive effect of propolis on the metabolic activity of periodontal ligament fibroblasts was reported and attributed to the increase in mitochondrial enzymatic activity [39,40]. Therefore, propolis extracts contain compounds, such as caffeic acid phenethyl ester [41], which is a chemopreventive agent of cellular oxidative stress [42]. The enhancement of cell growth using propolis extracts has been attributed to the increased expression of extracellular matrixes, including proteoglycans, glycosaminoglycan, elastin, and collagen [14]. Further studies are necessary to determine the biochemical processes influenced by the propolis nanoparticles, which in turn enhances the fibroblast cell metabolic activity.

## 4. Conclusions

The influence of propolis nanoparticles on the morphology and physicochemical properties of the collagen scaffolds were studied. A homogeneous distribution of the propolis NPs on the collagen surface was achieved using a propolis nanoparticle aqueous suspension. The impregnation of the collagen by NPs produced a change in the collagen D-pattern gap from 67 to 135 nm but did not affect the general fiber microstructure pattern, which suggests a good interaction and distribution of the particles. On the other hand, the interaction between the propolis nanoparticles and the collagen molecules influenced the collagen protein intermolecular cross-linking, which led to a decrease in the denaturation temperature and tensile strength when the concentration was above 100 μg/mL. The 3T3 fibroblast cell viability was higher when impregnated with the highest concentration of propolis NPs but the cell proliferation and percentage of living cells decreased in comparison with the pristine collagen. On the other hand, concentrations of 10 and 100 μg/mL enhanced the cell proliferation and cell survival for 24 h. A concentration of 100 μg/mL is suggested as optimal concentration to maintain the collagen structural and thermal properties, improve the cell viability, and enhance cell proliferation. Collagen–propolis NP films could become an affordable material used to produce bandages, coatings, and artificial tissue grafts that promote cell growth for healing wounds and skin diseases, with enhanced antimicrobial properties. Furthermore, the generated composite biomaterial can be adapted to specific uses, providing optimal scaffolding for its use in tissue engineering and regenerative medicine. We recommend impregnating other types of collagen scaffolds that promote metabolic activity to elucidate the synergic effects between the matrix and the NPs.

## Figures and Tables

**Table 1:** Summary of the denaturation temperature and enthalpy of collagen with different concentrations of nanoparticles calculated from the DSC curves. Clg = collagen scaffold. The enthalpy values were calculated as the integrated area under the peak divided by the mass of the sample.

Samples	Denaturation Temperature Td (°C)	Enthalpy ΔH (J/g)
Clg + propolis NPs 10 μg/mL	77.35	326.2
Clg + propolis NPs 100 μg/mL	63.10	209.8
Clg + propolis NPs 1000 μg/mL	63.50	213.0
Self-assembled Clg	81.52	446.6

## Supplementary Materials

The following are available online at https://www.mdpi.com/1996-1944/13/14/3118/s1, Figure S1: AFM (A) amplitude, (B) phase image of the collagen control sample. (C) Cross- section of the line profile shown in A. The collagen D-pattern gap is similar in all the microfibers measured and all the microfibers show a homogeneous phase.

## References

[1] Reyes F. (2014). Apósito Para Cicatrización de Heridas Comprometidas. España Patent.

[2] Naik G., Priyadarsini K., Satav J., Banavalikar M., Sohoni D., Biyani M., Mohan H. (2003). Comparative antioxidant activity of individual herbal. Phytochemistry.

[3] Rodeiro I., Donato M.T., Jimenez N., Garrido G., Molina-Torres J., Menendez R., Castell J.V., Gómez-Lechón M.J. (2009). Inhibition of Human P450 Enzymes by Natural. Phitotherapy Res..

[4] Castaldo S., Capasso F. (2002). Propolis, an old remedy used in modern medicine. Fitoterapia.

[5] Kuropatnicki A.K., Szliszka E., Krol W. (2013). Historical Aspects of Propolis Research in Modern Times. Evid. Based Complement. Altern. Med..

[6] Silva J.C., Rodrigues S., Feás X., Estevinho L. (2012). Antimicrobial activity, phenolic profile and role in the inflammation of propolis. Food Chem. Toxicol..

[7] Sulaiman G.M., al Sammarrae K.W., Ad’hiah A.H., Zucchetti M., Frapolli R., Bello E., Erba E., D’Incalci M., Bagnati R. (2011). Chemical characterization of Iraqi propolis samples and assessing their antioxidant potentials. Food Chem. Toxicol..

[8] Sodagar A., Akhavan A., Arab S., Bahador A., Pourhajibagher M., Soudi A. (2019). Evaluation of the Effect of Propolis Nanoparticles on Antimicrobial Properties and Shear Bond Strength of Orthodontic Composite Bonded to Bovine Enamel. Front. Dent..

[9] Mocanu A., Isopencu G., Busuioc C., Popa O.M., Socaciu-Siebert L. (2019). Bacterial cellulose films with ZnO nanoparticles and propolis extracts: Synergistic antimicrobial effect. Sci. Rep..

[10] Machado G.T.P., Veleirinho M.B., Mazzarino L., Filho L.C.P.M., Cerri M.M.R.L.A., Kuhnen S. (2019). Development of propolis nanoparticles for the treatment. Can. J. Anim. Sci..

[11] Kazemi F., Divsalar A., Saboury A.A., Seyedarabi A. (2019). Propolis nanoparticles prevent structural changes in human hemoglobin. Colloids Surf. B Biointerfaces.

[12] Dota K.F.D., Consolaro M.E.L., Svidzinski T.I.E., Bruschi M.L. (2011). Antifungal Activity of Brazilian Propolis Microparticles Against. Evid. -Based Complement. Altern. Med..

[13] Athikomkulchai S., Awale S., Ruangrungsi N., Ruchirawat S., Kadota S. (2013). Chemical constituents of Thai propolis. Fitoterapia.

[14] Olczyk P., Wisowski G., Komosinska-Vassev K., Stojko J., Klimek K., Olczyk M., Kozma E.M. (2013). Propolis Modifies Collagen Types I and III Accumulation in the Matrix of Burnt Tissue. Evid. Based Complement. Altern. Med..

[15] Hozzein W.N., Badr G., al Ghamdi A.A., Sayed A., Al-Waili N.S., Garraud O. (2015). Topical Application of Propolis Enhances Cutaneous Wound Healing by Promoting TGF-Beta/Smad-Mediated Collagen Production in a Streptozotocin-Induced Type I Diabetic Mouse Model. Cell. Physiol. Biochem..

[16] de Almeida E.B., Cardoso J.C., de Lima A.K., de Oliveira N.L., de Pontes-Filho N.T., Lima S.O., Souza I.C.L., de Albuquerque-Júnior R.L.C. (2013). The incorporation of Brazilian propolis into collagen-based dressing. J. Ethnopharmacol..

[17] Rassu G., Cossu M., Langasco R., Carta A., Giunchedi R.C., Gavini E. (2015). Propolis as lipid bioactive nano-carrier for topical nasal drug delivery. Colloids Surf. B Biointerfaces.

[18] Mello B.C., Petrus J.C.C., Hubinger M.D. (2010). Concentration of flavonoids and phenolic compounds in aqueous and ethanolic propolis extracts through nanofiltration. J. Food Eng..

[19] Marcucci M. (1995). Propolis: Chemical composition, biological properties and therapeutic activity. Apidologie.

[20] Wang K., Zhang J., Ping S., Ma Q., Chen X., Xuan H., Shi J., Zhanga C., Hu F. (2014). Anti-inflammatory effects of ethanol extracts of Chinese propolis and buds from poplar (Populus × canadensis). J. Ethnopharmacol..

[21] Rajan N., Habermehl J., Cote M.-F., Doillon C.J., Mantovani D. (2006). Preparation of ready-to-use, storable and reconstituted type I collagen from rat tail tendon for issue engineering aplications. Nat. Protoc..

[22] González-Masís J., Cubero-Sesin J., Vega-Baudrit J.R., González-Paz R.J. (2017). Development and characterization of biomaterials for biomimetic tissue applications. J. Eng. Med. Devices.

[23] O’brien J., Wilson I., Orton T., Pognan F. (2000). Investigation of the Alamar Blue (resazurin) fluorescent dye for the assessment of mammalian cell cytotoxicity. Eur. J. Biochem..

[24] Pobiega K., Kra’sniewska K., Derewiaka D., Gniewosz M. (2019). Comparison of the antimicrobial activity of propolis extracts. J. Food Sci. Technol..

[25] Skliar M., Chernyshev V.S. (2019). Imaging of extracellular vesicles by atomic force microcopy. J. Visualized Exp..

[26] Yusof N.S.M., Ashokkumar M. (2015). Ultrasonic Modification of Micelle Structures. Handbook of Ultrasonics and Sonochemistry.

[27] Kubiliene L., Laugaliene V., Pavilonis A., Maruska A., Majiene D., Barcauskaite K., Kubilius R., Kasparaviciene G., Savickas A. (2015). Alternative preparation of propolis extracts: Comparison of their composition and biological activities. BMC Complement. Altern. Med..

[28] Do Nascimento T.G., Da Silva P.F., Azevedo L.F., Da Rocha L.G., de Moraes Porto I.C., e Moura T.F., Basílio-Júnior I.D., Grillo L.A., Dornelas C.B., da Silva Fonseca E.J. (2016). Polymeric Nanoparticles of Brazilian Red Propolis Extract: Preparation, Characterization, Antioxidant and Leishmanicidal Activity. Nanosc. Res. Lett..

[29] Liu X., Dan N., Dan W. (2017). Insight into the Collagen Assembly in the Presence of Lysine and Glutamic Acid: An in Vitro Study. Mater. Sci. Eng..

[30] Ferreira A., González G., González-Paz R., Feijoo J., Lira-Olivares J., Noris-Suárez K. (2009). Bone collagen role in piezoelectric mediated remineralization. Acta Microsc..

[31] Kim H.G., Kim J.H. (2011). Preparation and Properties of Antibacterial Poly (vinyl alcohol). Fibers Polym..

[32] Miles C.A., Burjanadze T.V., Bailey A.J. (1995). The Kinetics of the Thermal Denaturation of Collagen in Unrestrained Rat Tail Tendon Determined by Differential Scanning Calorimetry. J. Mol. Biol..

[33] M F., Banaszak M. (2013). Variation in type I collagen fibril nanomorphology: The significance and origin. Bone Key Rep..

[34] Krell R. (1996). Value-Added Products from Beekeeping.

[35] Yousefi M., Ariffin F.A., Huda N. (2016). An alternative source of type I collagen based on by-product with higer thermal stability. Food Hydrocolloids.

[36] Tyszka-Czochara M., Paśko P., Reczyński W., Szlósarczyk M., Bystrowska B., Opoka W. (2014). Zinc and Propolis Reduces Cytotoxicity and Proliferation in Skin Fibroblast Cell Culture: Total Polyphenol Content and Antioxidant Capacity of Propolis. Biol. Trace Elem. Res..

[37] Elkhenany H., El-Badri N., Dhar M. (2019). Green propolis extract promotes in vitro proliferation, differentiation, and migration of bone marrow stromal cells. Biomed. Pharmacother..

[38] Fung C.S., Mohamad H., Hashim S.N., Htun A.T., Ahmad A. (2015). Proliferative Effect of Malaysian Propolis on Stem Cells from Human Exfoliated Deciduous Teeth: An in vitroStudy. Br. J. Pharm. Res..

[39] Gjertsen A.W., Stothz K.A., Neiva K.G., Pileggi R. (2011). Effect of propolis on proliferation and apoptosis of periodontal ligament fibroblasts. Oral Surg. Oral Med. Oral Pathol. Oral Radiol. Endodontol..

[40] Grenho L., Barro J., Ferreira C., Santos V.R., Monteiro F.J., Ferraz M., Cortes M.E. (2015). In vitro antimicrobial activity and biocompatibility of propolis containing nanohydroxyapatite. Biomed. Mater..

[41] Grunberger D., Banerjee R., Eisinger K., Oltz E.M., Efros L., Caldwell M., Estevez V., Nakanishi K. (1988). Preferential cytotoxicity on tumor cells by caffeic acid phenethyl ester isolated from propolis. Experientia.

[42] Frenkel K., Wei H., Bhimani R., Ye J., Zadunaisky J., Huang M.T., Ferraro T., Conney A.H., Grunberger D. (1993). Inhibition of Tumor Promoter-Mediated Processes in Mouse Skin and Bovine Lens by Caffeic Acid Phenethyl Ester. Cancer Res..

