# Supplementary material for: Increased Fibroblast Metabolic Activity of Collagen Scaffolds via the Addition of Propolis Nanoparticles
Source: Materials (Basel). 2020 Jul 13;13(14):3118. doi: 10.3390/ma13143118 (PMC7412245; doi:10.3390/ma13143118)
Supplement: Supplementary file 1 [file materials-13-03118-s001.pdf]

materials

Supplementary Information

Increased Fibroblast Metabolic Activity of Collagen Scaffolds via the Addition of Propolis Nanoparticles

Jeimmy González-Masís [1], Jorge M. Cubero-Sesin [1], Yendry R. Corrales-Ureña [2], Sara González-Camacho [3], Nohelia Mora-Ugalde [4], Mónica Baizán-Rojas [4], Randall Loaiza [4], José Roberto Vega-Baudrit [2,5] and Rodolfo J. Gonzalez-Paz [2,*]

[1] Centro de Investigación y Extensión en Materiales, Escuela de Ciencia e Ingeniería de los Materiales, Instituto Tecnológico de Costa Rica, 159-7050 Cartago, Costa Rica; jeimygonz@gmail.com (J.G.-M.); jorgecubero@gmail.com (J.M.C.-S.)
[2] Adolphe Merkle Institute, University of Fribourg, Chemin des Verdiers 4, 1700 Fribourg, Switzerland; yendry386@hotmail.com (Y.R.C.-U.); jvegab@gmail.com (J.R.V.-B.)
[3] Biological Assay Laboratory (LEBi), Universidad de Costa Rica, 11501-2060 San José, Costa Rica; sara.gonzalez@ucr.ac.cr
[4] National Center for Biotechnological Innovations (CENIBiot), National Center of High Technology (CeNAT-CONARE), Pavas, 1174-1200 San José, Costa Rica; none.0512@gmail.com (N.M.-U.); mbaizan@cenat.ac.cr (M.B.-R.); rloaiza@cenat.ac.cr (R.L.)
[5] National Laboratory of Nanotechnology (LANOTEC), National Center of High Technology (LANOTEC-CeNAT-CONARE), Pavas, 1174-1200 San José, Costa Rica
[6] Laboratorio de Polímeros (POLIUNA), Universidad Nacional, 86-3000 Heredia, Costa Rica
* Correspondence: osarsip@gmail.com

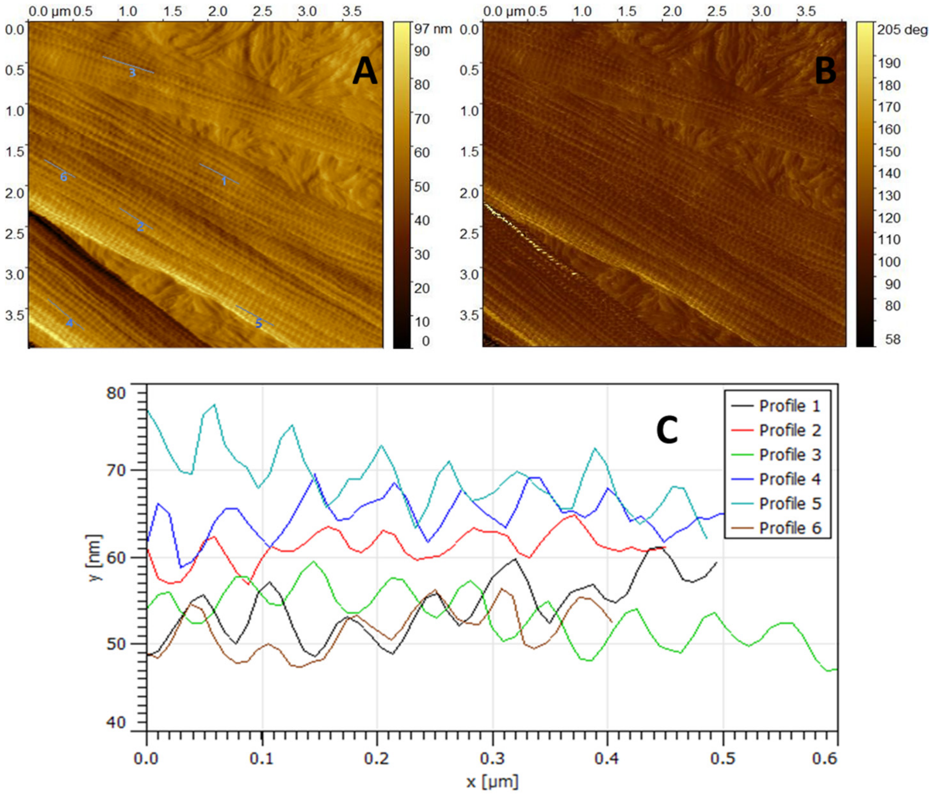

Figure S1. AFM (**A**) amplitude, (**B**) phase image of the collagen control sample. (**C**) Cross- section of the line profile shown in A. The collagen D-pattern gap is similar in all the microfibers measured and all the microfibers show a homogeneous phase.

© 2020 by the authors. Licensee MDPI, Basel, Switzerland. This article is an open access article distributed under the terms and conditions of the Creative Commons Attribution (CC BY) license (http://creativecommons.org/licenses/by/4.0/).
